# Accident injuries of adults in Germany

**DOI:** 10.17886/RKI-GBE-2017-072

**Published:** 2017-10-09

**Authors:** Anke-Christine Saß, Ronny Kuhnert, Alexander Rommel

**Affiliations:** Robert Koch Institute, Department of Epidemiology and Health Monitoring, Berlin, Germany

**Keywords:** ACCIDENT, INJURY, ADULTS, HEALTH MONITORING, GERMANY

## Abstract

In 2014, according to estimates by the Federal Institute for Occupational Safety and Health (BAuA), around 9.8 million people in Germany suffered accident injuries. Over 22,000 people died. Federal statistics, however, cannot comprehensively describe accidents in Germany. Here, the Robert Koch Institute health surveys provide an important addition. In the GEDA 2014/2015-EHIS survey, 10.5% of men and 6.9% of women reported that they had suffered accident injuries requiring medical treatment during the past 12 months. Young men aged 18 to 29 have the highest accident risk (18.1%). The overall accident injury figures have hardly changed since the previous GEDA 2012 survey. Preventing accidents is a highly important topic not only for the victims of accidents and their families, but also for society as a whole. According to the World Health Organization, a largely untapped potential for accident prevention remains.

## Introduction

Preventing accidents is highly important, both for the victims of accidents and their families and also for society as a whole. According to the Federal Institute for Occupational Safety and Health (BAuA) estimates, around 9.8 million people suffered accident injuries in 2014 [1]. More than 22,000 people died in accidents (ICD-10: V01-X59; ICD-10: International Statistical Classification of Diseases and Related Health Problems, 10th revision) [2]. Besides unintentional accident injuries, injuries can also be caused intentionally, for example during fights (interpersonal violence such as attacks or brawls) or in cases of intentional self-harm. The World Health Organization (WHO) estimates that 72% of all injuries in Europe are unintentional [3]. The medical treatment of injuries accounts for around 5% of total annual medical expenses (ICD-10: S00-T98; 2008) [4]. In 2015, around 11% of incapacity days for economically active members of the AOK health insurance fund were related to injuries [5]. Nearly every 10th premature death (death before the age of 65) in 2015 was due to injury [6].

Official statistics, however, cannot provide a comprehensive picture of accidents in Germany (see info box) [7]. Fatal accidents are recorded in cause of death statistics. Occupational accidents are documented comprehensively by the corresponding accident insurance providers; traffic accidents are recorded in road accident statistics. These statistics, however, fail to provide systematic data on further important fields such as accidents that occur at home, during leisure time or those road accidents where the police is not involved. Representative surveys (such as health surveys) can provide additional information and ensure a clearer overall picture of non-fatal accidents in Germany. They are therefore an important supplement [7].

**Info box:**

Official German statistics collect data on injuries based on the ICD classifications:

Chapter XIX (S00-T98) Injury, poisoning and certain other consequences of external causes

► The affected part of the body and type of injury is coded; no distinction is made between intentional and unintentional injuries (accidents)

Chapter XX (V01-Y98) External causes of morbidity and mortality

► Differentiation between intentional and unintentional injuries (accidents) is possible; is only used to codify causes of death (cause of death statistics)

Numerous health surveys which the Robert Koch Institute (RKI) conducted during the past few years provide cross-sectional and time series data on accidents for adults (and children) in Germany. The GEDA 2014/2015-EHIS survey provides current data. This survey integrated the European Health Interview Survey (EHIS) into the German Health Update survey (GEDA 2014/2015), which also involved substantial changes to the way in which questions are posed. This created challenges regarding numerous health topics as well as for non-fatal accidents mainly because, whilst GEDA 2014/2015-EHIS aims to ensure the comparability of findings across Europe, the survey also hopes to provide relevant data for preventive healthcare and health policy in Germany.

## Indicator

GEDA 2014/2015-EHIS surveyed accident injuries through self-administered paper-based or online questionnaires. The first question was, ‘During the past 12 months have you suffered an injury from one of the following accidents? This includes injuries caused by poisoning, animals or insects. Injuries caused intentionally by other people are excluded.’ The available answer categories were ‘Road accident’, ‘Accident at home’, and ‘Accident during leisure time’.

All of those who had suffered an accident were then asked whether their injuries had required medical treatment, ‘Following your accident, did you require medical attention? If you suffered several accidents, this question refers to your severest accident, i.e. the accident that required the most medical care.’ Respondents could answer, ‘Care in hospital or other healthcare facility as inpatient or outpatient’, ‘Care provided by physician or nurse’, ‘No medical attention’.

GEDA 2014/2015-EHIS supplemented both of these questions, which form part of the EHIS instrument, with questions on occupational accidents so as to include accidents at work in the overall picture of accidents in Germany. Commuting accidents, i.e. accidents that occur on the way to work, were excluded. Insurance legislation in Germany covers these accidents through occupational accident insurance. Respondents were asked about occupational accidents and medical attention for resulting injuries by asking, ‘Did you suffer an injury at work during the past 12 months? This does not include commuting accidents’, and, ‘Did you require medical attention for your occupational accident?’ Possible answers were ‘Yes’ or ‘No’.

To calculate the indicator ‘Accident injury requiring medical attention’, a combination of the questions asked was used. To enter this category, a person had to have suffered a road accident, an accident at home or during leisure time and then sought medical attention in a hospital or other medical facility either as an inpatient or outpatient. Respondents who had suffered an occupational accident that had required medical attention were also included. Such a definition provides an overview of all non-fatal accidents in Germany (excluding minor injuries). Moreover, this approach allows us to continue the already established RKI time series. The results should be seen as estimates, as they combine two not fully identical questions/answers.

**GEDA 2014/2015-EHIS**

**Data holder:** Robert Koch Institute

**Aims:** To provide reliable information about the population’s health status, health-related behaviour and health care in Germany, with the possibility of a European comparison

**Method:** Questionnaires completed on paper or online

**Population:** People aged 18 years and above with permanent residency in Germany

**Sampling:** Registry office sample; randomly selected individuals from 301 communities in Germany were invited to participate

**Participants:** 24,016 people (13,144 women; 10,872 men)

**Response rate:** 26.9%

**Study period:** November 2014 - July 2015

**Data protection:** This study was undertaken in strict accordance with the data protection regulations set out in the German Federal Data Protection Act and was approved by the German Federal Commissioner for Data Protection and Freedom of Information. Participation in the study was voluntary. The participants were fully informed about the study’s aims and content, and about data protection. All participants provided written informed consent.

More information in German is available at

www.geda-studie.de

The results are stratified by gender, age and education. The International Standard Classification of Education (ISCED) was used to classify the responses provided on educational level [8]. Differences between these groups are interpreted as statistically significant if the respective confidence intervals do not overlap.

The analyses are based on the data from 23,147 participants aged 18 years and above (12,625 women, 10,522 men) with valid data on road and occupational accidents as well as accidents at home and during leisure time. The calculations were carried out using a weighting factor that corrects for deviations within the sample from the German population (as of 31 December 2014) with regard to gender, age, district type and education. The district type reflects the degree of urbanisation and accounts for the regional distribution in Germany.

For a detailed description of the methodology applied in the GEDA 2014/2015-EHIS study, see Lange et al. 2017 [9] as well as the article German Health Update: New data for Germany and Europe in Issue 1/2017 of the Journal of Health Monitoring. This includes the questionnaire which was used in the survey.

## Results and discussion

As reported by GEDA 2014/2015-EHIS, 8.6% of adults in Germany suffered injuries from accidents during the past 12 months that required medical treatment (accident prevalence). Prevalence for men was 10.5% and slightly lower for women at 6.9% (Table 1). Compared to GEDA 2012, accident injury rates in GEDA 2014/2015-EHIS remained nearly unchanged (8.7% in 2012) [10]. Accident prevalence rates by gender also remained nearly constant. The survey registered a marginal decrease in the rate for men and a slight increase for women (rates of 10.9% and 6.6% respectively for accident injuries in 2012).

Stratified by age and gender, the group of young men aged 18 to 29 particularly stands out. Presenting an 18.1% accident injury rate over the past 12 months, this group is involved particularly often in accidents that require medical attention. Accident prevalence among men decreases with age, but up to the 45- to 64-year age group remains significantly higher than for women. The rate for men aged 65 and above then drops to 5.3%. Compared to the other age groups, the rates are significantly higher for the youngest group and significantly lower for the oldest group.

Accident prevalence for young women is also higher, yet lower than for men. 9.8% of women aged 18 to 29 suffer accident injuries. These rates for women drop at middle age, but, unlike men, begin to increase again at age 65. In this age group, 8.5% suffer accident injuries. The figures for the youngest and oldest age group are therefore significantly higher than for the other age groups.

Riskier behaviour (risk seeking behaviour) is referred to as an explanation for the observed gender differences (and the particularly high accident prevalence among young men) [11]. Further differences, in which gender also plays a role, exist for the prevalence of occupational accidents. In numerous male dominated professions, the accident risk is simply higher, such as in the building sector [11].

Compared to GEDA 2012, age and gender patterns for the distribution of accident injuries are similar. However, this survey did not record an increase in accident prevalence for women aged 65 and above [10]. The only comprehensive survey of accidents and injuries so far was conducted in 2010 in the context of the accident module (GEDA 2010). The results of this survey, which the RKI published in the Contributions to Federal Health Reporting series, provided detailed insights on the accidents suffered by adults [7]. Like the current GEDA survey, the GEDA 2010 accident module already hinted at this tendency of accident rates among older women to increase again [7]. By surveying the type of accidents women suffered, it became clear that women aged 65 and above are more likely to suffer falls than men. Compared to men, the results from hospital diagnosis statistics also indicate an increased risk of injury for women aged 65 and above (inpatient treatment of injuries classified under ICD-10: S00-T98) [12].

Concerning education, GEDA 2014/2015-EHIS reveals a slightly lower accident prevalence among women and men from a high education background (Table 1). However, this does not apply equally to all age groups. The accident module in GEDA 2010 provided a more differentiated picture: people of high socio-economic status tended to report more accidents in leisure time, whereas people with low social status more frequently reported occupational accidents [7]. For the overall prevalence of accident by social status, however, no differences were recorded. Conclusive evidence supports the existence of this strong correlation between the risk of suffering occupational accidents and social status: Employees in high status jobs significantly less often suffer accidents than those working in low status jobs. Mainly, this is due to the fact that the latter occupations more often involve hazardous work [13, 14].

GEDA 2014/2015-EHIS also provides regional prevalence data by federal state. The information system of Federal Health Reporting (www.gbe-bund.de, in German) provides data on accident prevalence by federal state, age and gender. There are only marginal differences in the prevalence of accidents between the German federal states.

Concerning the place of accident, GEDA 2014/2015-EHIS reveals that most non-fatal accidents occur at home or during leisure time. 6.4% of women (CI 5.9-6.9) and 6.2% of men (CI 5.7-6.8) reported accidents at home. 7.3% of women (CI 6.7-7.9) and 10.8% of men (CI 10.0-11.7) reported accidents during leisure time. Due to the format of the corresponding questions in the EHIS questionnaire, no conclusions can be drawn on the percentage of these accidents that required medical treatment. For fatal accidents too, cause of death statistics indicate that 80% occur at home or during leisure time [2]. Current GEDA survey results are therefore in line with earlier GEDA survey waves as well as with the annual estimates of Germany’s Federal Institute for Occupational Safety and Health (BAuA) [1, 7, 10, 15, 16]. In GEDA, the workplace figures as the third most frequent place of accident and a significantly higher number of men suffer occupational accidents than women (5.9% vs. 2.7%; CI 5.4-6.6 and 2.3-3.1).

A comparison of accident prevalence as reported in GEDA 2014/2015-EHIS with other data sources is not fully possible. This is due to the differences in survey methods and content, for example that official hospital diagnosis statistics do not differentiate between unintentional and intentional injuries. BAuA annually estimates total accident prevalence in Germany and does also differentiate according to places of accident. The current estimate reports that roughly 12% of the population suffer accident injuries per year [1]. GEDA 2014/2015-EHIS reports a lower prevalence of just under 9% for adults. BAuA figures, however, also include children and numerous surveys indicate that children are more likely to suffer accidents than adults [17-19].

In spite of highly positive developments, such as the significant decrease in fatal road accidents during the past 20 years [20], accident injuries remain a prime public health concern, regarding not only the prevalence of accidents, their sometimes dire consequences and related high costs of treatment, but also concerning prevention: The WHO considers that most intentional and unintentional injuries could be prevented [3].

## Key statements

- 11% of men and 7% of women suffered accident injuries requiring medical treatment during the past 12 months.
- Young men aged 18 to 29 presented the highest risk of suffering an accident (18% are accident victims during the past 12 months).
- The share of accident victims has remained constant since the previous GEDA survey: 9% (2012 and 2014/2015).
- Most (non-fatal and fatal) accidents occur at home and during leisure time.

## Figures and Tables

**Table 1:** 12-month prevalence of accidents requiring medical treatment according to gender, age and educational level (n=12,625 women; n=10,522 men) Source: GEDA 2014/2015-EHIS

Women	%	(95% CI)	Men	%	(95% CI)
**Women total**	**6.9**	**(6.3-7.4)**	**Men total**	**10.5**	**(9.7-11.3)**
**18-29 Years**	9.8	(8.3-11.7)	**18-29 Years**	18.1	(15.6-20.9)
Low education	13.2	(9.4-18.3)	Low education	23.1	(17.3-30.1)
Medium education	10.0	(8.1-12.3)	Medium education	17.2	(14.2-20.8)
High education	3.7	(2.1-6.7)	High education	13.1	(9.2-18.2)
**30-44 Years**	5.3	(4.3-6.4)	**30-44 Years**	11.0	(9.6-12.7)
Low education	9.2	(5.5-15.2)	Low education	11.9	(7.7-18.0)
Medium education	5.4	(4.1-7.0)	Medium education	13.8	(11.4-16.4)
High education	2.9	(2.1-4.0)	High education	5.9	(4.4-7.7)
**45-64 Years**	5.3	(4.6-6.1)	**45-64 Years**	9.5	(8.3-10.8)
Low education	6.0	(4.1-8.6)	Low education	11.3	(8.4-14.9)
Medium education	5.3	(4.5-6.3)	Medium education	11.1	(9.4-13.1)
High education	4.2	(3.2-5.6)	High education	5.9	(4.8-7.1)
**≥ 65 Years**	8.5	(7.3-10.0)	**≥ 65 Years**	5.3	(4.3-6.5)
Low education	8.9	(6.7-11.6)	Low education	5.6	(3.4-8.9)
Medium education	8.5	(6.8-10.5)	Medium education	4.0	(2.9-5.5)
High education	7.2	(4.7-10.7)	High education	7.5	(5.5-10.2)
**Total (women and men)**	**8.6**	**(8.2-9.2)**	**Total (women and men)**	**8.6**	**(8.2-9.2)**

CI=Confidence interval
